# Pharmacogenomics of MDD as a Developing Field: Challenges and Opportunities

**DOI:** 10.1192/j.eurpsy.2022.136

**Published:** 2022-09-01

**Authors:** B. Baune

**Affiliations:** Westfaelische-Wilhelms-University Muenster (WWU), Department Of Mental Health, Muenster, Germany

**Keywords:** MDD, pharmacogenomics, Transcriptomics, polygenic risk scores

## Abstract

While first gene-drug pairs have emerged to be clinically actionable in the treatment of major depressive disorders (MDD) (e.g., CYP2D6 and TCAs/SSRIs), genomic studies have not yet been successful in identifying replicable and valid biomarkers of pharmacological treatment outcome. While some trials suggest that candidates such as CYP2D6, CYP2C19, CYP1A2, SLC6A4 and HTR2A polymorphisms may improve the prediction of response/remission, these results should be interpreted cautiously and required confirmation in larger samples. This presentation will cover state of the art of pharmacogenomics for MDD as well as the emerging field of pharmacotranscriptomics and functional genomics analyses in MDD. Specifically, pharmacotranscriptomics in combination with genomics may be a promising avenue in overcoming some of the current limitations in treatment response prediction research. More recently, the combined genetic effect of polygenic risk scores has shown promising results in predicting treatment response. Importantly, adequately large and well phenotyped clinical trials are required to be conducted with pharmacogenomics/-transcriptomics prospectively in mind.

**Disclosure:**

No significant relationships.

